# Particle-Based Microfluidic Quartz Crystal Microbalance (QCM) Biosensing Utilizing Mass Amplification and Magnetic Bead Convection

**DOI:** 10.3390/mi9040194

**Published:** 2018-04-18

**Authors:** Jan-W. Thies, Bettina Thürmann, Anke Vierheller, Andreas Dietzel

**Affiliations:** 1 Institute of Microtechnology (IMT), TU Braunschweig, Alte Salzdahlumer Str. 203, 38124 Braunschweig, Germany; j.thies@tu-braunschweig.de (J.-W.T.); b.thuermann@tu-braunschweig.de (B.T.); a.vierheller@tu-braunschweig.de (A.V.); 2 Center of Pharmaceutical Engineering (PVZ), TU Braunschweig, Franz-Liszt-Straße 35 A, 38106 Braunschweig, Germany

**Keywords:** quartz crystal microbalance (QCM), sensitivity enhancement, superparamagnetic particles, magnetic manipulation, lab-on-a-chip

## Abstract

Microfluidic quartz crystal microbalances (QCM) can be used as powerful biosensors that not only allow quantifying a target analyte, but also provide kinetic information about the surface processes of binding and release. Nevertheless, their practical use as point-of-care devices is restricted by a limit of detection (LoD) of some ng/cm². It prohibits the measurement of small molecules in low concentrations within the initial sample. Here, two concepts based on superparamagnetic particles are presented that allow enhancing the LoD of a QCM. First, a particle-enhanced C-reactive protein (CRP) measurement on a QCM is shown. The signal response could be increased by a factor of up to five by utilizing the particles for mass amplification. Further, a scheme for sample pre-preparation utilizing convective up-concentration involving magnetic bead manipulation is investigated. These experiments are carried out with a glass device that is fabricated by utilizing a femtosecond laser. Operation regimes for the magnetic manipulation of particles within the microfluidic channel with integrated pole pieces that are activated by external permanent magnets are described. Finally, the potential combination of the concepts of mass amplification and up-concentration within an integrated lab-on-a chip device is discussed.

## 1. Introduction

Quartz crystal microbalance (QCM) sensors are measuring very small changes of mass adsorbed at the surface of an oscillating quartz crystal disc as a resonance frequency change. In combination with specialized coatings, these sensors are able to measure and quantify a broad spectrum of different substances, including biologically relevant molecules or even entire viruses, bacteria, or cells. Since this technology has already been known for nearly six decades, a vast amount of applications have been reported. A good overview over QCM biosensor applications is given in [1,2,3].

As a rule of thumb, a limit of detection (LoD) around some ng/cm^2^ can be reached with QCMs [4,5]. This allows measuring a lot of different substances within blood samples, such as the acute phase protein C-reactive protein (CRP, molecular weight (MW) around 115.135 Da). Its concentration in blood rises from 1 mg/mL to 10 mg/mL in a healthy state, and rises up to 200 mg/mL after severe inflammation events. The CRP level allows discriminating between viral and bacterial infections, and therefore is very helpful for treatment decisions. However, the serum level of CRP alone will not provide diagnostic information. Nevertheless, it is a useful parameter and an early indicator because its concentration increases within 24 h after inflammation events [6]. For smaller proteins with lower molecular masses—including peptide hormones such as insulin (MW around 5.808 Da), which has a concentration in blood between 50–250 pmol/L (equal to 0.29–1.45 µg/L) and inhibits a key function in the regulation of the glucose level within blood [7]—the limit of detection is not sufficient without signal enhancement techniques.

Besides constructive modifications of QCM for improving sensitivity, mass enhancement by the attachment of additional molecules or particles is also an option. These additional molecules and/or particles bind to the substance to be measured, which results in a bigger frequency change [8]. In a previous work [9], we utilized an especially designed CRP antibody sandwich with a fast off-rate. There, the dissociation of the CRP complex happened spontaneously without the need for additional elution steps, allowing for faster analysis and reducing the consumption of chemicals. However, an additional particle attachment was not stable enough to work step-by-step, because dissociation took place before the particles arrived at the QCM. As a result, we utilize a different superparamagnetic particle-enhanced sandwich in the following.

Furthermore, if superparamagnetic particles are used for mass enhancement, their magnetic properties allow their manipulation through the use of external magnets. The particles can be moved through the sample to meet more target analyte molecules that bind to their surface, leading to an up-concentration of target analyte on the particles. Previously [10], a manipulation system was fabricated utilizing electroplated pole pieces made from a soft magnetic NiFe alloy on a glass substrate and a polydimethylsiloxane (PDMS) cover containing the fluidic channel and the cavities for the pole pieces. Since the utilized electroplating process involved certain problems, such as irregular deposition rates and leakage along the small PDMS lid between the pole pieces, the whole fabrication process was reworked. A new fs-laser process was developed that allows the fabrication of the manipulation system solely from glass, while the pole piece material is freely selectable. Leakage between the fluidic channel and pole piece cavities (distance 200 µm) is avoided using a thermal bonding process.

In the following, our microfluidic QCM device is introduced together with the process of mass enhancement by particle application. Further, the design and fabrication of a simple but efficient particle manipulation device is presented. For the operation of the magnetic manipulation system, only low-cost permanent magnets are used to activate the embedded pole pieces structures. Finally, a concept for combining both devices is discussed.

## 2. Materials and Methods

### 2.1. QCM Signal Amplification Using Particles

In 1959, Sauerbrey [4] described the relationship between the change of the fundamental characteristic resonant frequency Δ*f*_m_ of an oscillating QCM due to an occurring mass change Δ*m*_s_ as:

(1) $$\Delta f_{m}=\frac{-f_{0}}{\rho_{Q}\cdot t_{Q}}\cdot \frac{\Delta m_{s}}{A_{el}}$$

where *f*_0_ is the characteristic resonant frequency, which is defined by the crystallographic orientation of the blank, *t_Q_* is the oscillator thickness, *A_el_* is the area of the electrodes on both sides of the quartz through which an alternating electrical potential is applied to the piezoelectric disc, and *ρ_Q_* = 2.65 × 10^3^ kg m^−3^ is the quartz density. This relationship is only strictly valid within a vacuum and for total mass increases below 2%.

The strength of the signal Δ*f_m_*, and therefore also the limit of detection of a QCM, is influenced by the oscillators’ constructive design parameters. According to Equation (1), a reduction of $t_{Q}$ [11] and an increase of $f_{0}$ improve the sensitivity of a QCM. This makes the miniaturization of such a device favorable. *f*_0_ can also be increased by operating the QCM at overtones [12]. For this approach, complex oscillation circuits are necessary. Nevertheless, some commercial QCM suppliers do utilize overtones within their oscillation equipment. $\Delta m_{s}$ can be increased by an increase of electrode area $A_{el}$, which is the surface where recognition molecules are immobilized. This approach would not lead to an increased $\Delta f_{m}$. However, $\Delta m_{s}$ can also be increased by adding weight to the bound target molecules, which is the strategy investigated here.

**Figure 1:**
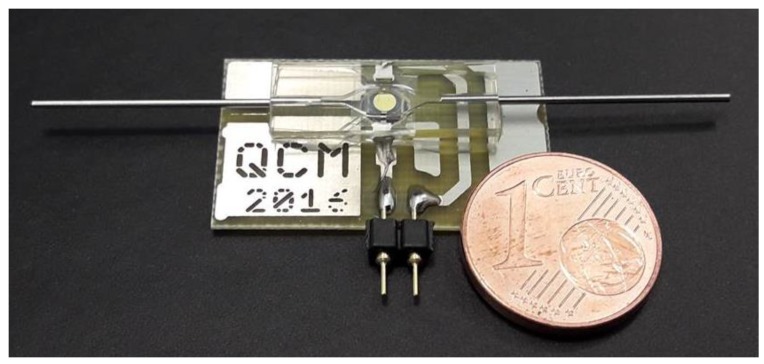
Flow cell device with integrated microfabricated quartz crystal microbalances (QCM) in comparison to a one-cent coin.

For this study, custom microfabricated QCM devices with flow cell embedding are made from PDMS (Figure 1), as described earlier [9]. For injecting fluids into the microsystem, a pulsation-free syringe pump (Nemesys syringe pump, Cetoni GmbH, Korbussen Germany) is used. To stimulate the QCM, a custom developed oscillation circuit with resonator control establishing a control loop is used to maintain oscillation, even when heavy damping occurs. This low-cost oscillation circuit is described in Beißner et al. [13] (open access), including its layout and part list as supplemental material, allowing its reproduction. 

**Figure 2:**
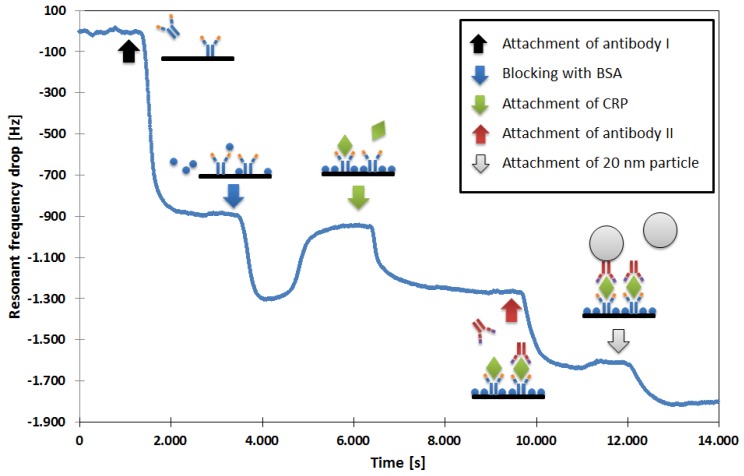
C-reactive protein (CRP) measurement utilizing the mass enhancement protocol involving a second antibody and streptavidin-coated superparamagnetic nanoparticles.

Our first aim was to improve the limit of a mass enhancement with additional molecules and particles instead of the further miniaturization of the QCM. We developed a protocol that can be understood on the basis of the measurement shown in Figure 2:

First, the sensor is coated with a self-assembled monolayer (SAM) to allow the attachment of detection antibodies to the QCM’s gold electrode [14]. This SAM is made in two steps. First, the chip is incubated with a cysteamine solution (Sigma-Aldrich, Darmstadt, Germany, 20 mmol/L in deionized water) for 12 h. Then, the flow cell is filled for two hours with glutaraldehyde (Sigma-Aldrich, 2.5 vol. % in Phosphate-buffered saline (PBS)), after which the chip is ready to use. While measuring, the flow cell is constantly purged with a PBS buffer solution. The samples are applied into the buffer through an injection valve (Model 7125, with 100-µL sample loop, Rheodyne, Rohnert Park, CA, USA). After the QCM has stabilized, the CRP detection antibody (MCA5880G; Bio-Rad Laboratories, Inc. Hercules, CA, USA; concentration 20 µg/mL) is applied, resulting in a persisting drop in resonant frequency when it gets attached to the QCM. To prevent unspecific binding, the remaining free gold surface and SAM binding sites are blocked with bovine serum albumin (BSA; Sigma-Aldrich, 1% (m/v) in deionized water) solution. This step is associated with a persistent and temporary drop in resonant frequency. While the persisting part is due to the BSA attachment, the temporary part is observable as long as the BSA solution is located within the flow cell. Since it has a higher viscosity than the PBS buffer, it dampens the QCM. This temporary change in resonant frequency disappears when the BSA solution leaves the flow cell. At this point, the sensor is prepared for the measurement of CRP.

The application of the sample (here containing 20 µg/mL CRP, Bio-Rad Laboratories, Inc., product code 1707–2029) results in a concentration-dependent frequency drop. As long as the signal is clearly distinguishable from background noise/fluctuations, no further measures have to be taken. In other cases (for lower CRP concentrations), the signal can then be increased by mass amplification. Analogous to the detection sandwiches that are used in many immunoassays [15], a second antibody (MCA5882G; Bio-Rad Laboratories, Inc.; concentration 20 µg/mL), which binds to a different CRP epitope, is applied. To allow for an even further mass increase, this antibody was labeled with biotin (LNK042B, Bio-Rad Laboratories, Inc.), and in an additional step, streptavidin-coated, 20-nm nanoparticles (Absolute Mag™ Streptavidin Magnetic Particles, 20 nm; Creative Diagnostics, New York, NY, USA) are applied. These superparamagnetic nanoparticles were labeled by the manufacturer, with approximately one molecule of streptavidin per particle.

The second antibody can only bind to the QCM when CRP is present, and the particles get bound to the biotin molecule of the second antibody. The observed resonant frequency drops can be considered CRP concentration-dependent signal amplification.

Of course, all of the involved substances can be also applied at once. After the measurement, the sensor can be regenerated by applying 0.1 M glycine hydrochloride (G2879-100G, Sigma-Aldrich) to elute the bound CRP and everything attached to it. Directly afterwards, the next CRP-containing sample can be analyzed.

Due to variations in the quality of the surface activation, calibration with a sample of known composition for determination of the QCM’s sensitivity is necessary in order to obtain comparable results.

### 2.2. Concept of the Magnetic Bead Convection System

Particle manipulation within microfluidic channels is investigated for a lot of different processes, including active mixing [16,17,18], controlled transportation [19], or increasing the active surface area for chemical or biological processes. A good overview over applications of superparamagnetic particles in microfluidic systems can be found in [20,21,22]. The desired particle motion is in many cases enabled by complex, external equipment for generation and dynamically controlling magnetic fields. A good overview over magnet systems and manipulation mechanisms can be found in the review of Cao et al. [23]. In contrast to these complex and costly systems, magnetic guiding structures can be used in combination with permanent magnets as a cheap and low-cost option for manipulation. For example, Adams et al. [24] utilized ferromagnetic strips for the sorting of cells labeled with different magnetic tags and non-labeled cells. While a permanent magnet was used for pulling the tagged cells near the ferromagnetic strips, the strips generated a short range magnetic field gradient. Here, we utilize a magnetic guidance structure for a convective up-concentration approach. It only requires one stationary, external permanent magnet to facilitate controlled movement in two directions [10], allowing easy integration into microfluidic measurement systems. Another advantage of utilizing a permanent magnet over an electromagnet of the same size is the stronger field that is generated.

To accelerate a spherical particle within a viscous medium, the hydrodynamic drag force ${\vec{F}}_{D}$ has to be overcome:

(2) $${\vec{F}}_{D}=-6\pi \eta r\vec{v}$$

Here, $\vec{v}$ is the particle’s velocity, r the particle radius, and *η* is the dynamic viscosity of the surrounding medium. For the magnetic force, ${\vec{F}}_{mag}$, which acts on a magnetic dipole moment $\vec{m}$ in a B-field $\vec{B}$. , can be described as:

(3) $${\vec{F}}_{mag}=\left(\vec{m}\nabla \right)\vec{B}.$$

**Figure 3:**
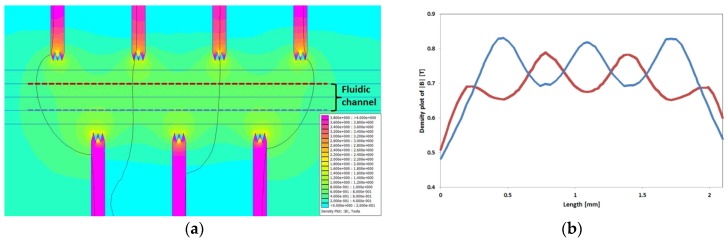
(**a**) Simulation of the magnetic field density within a microfluidic channel surrounded by seven interlocked pole pieces exposed to the field of a one permanent magnet. (**b**) Progress of the magnetic field density along the blue and red line in Figure 3a. Colors in both figures indicate their relation. The final magnetic manipulation system is shown in Section 3.2.

The force is proportional to the field gradient; hence, particle manipulation within a homogeneous field is impossible. For this reason, we have introduced soft magnetic flux concentration structures along our microfluidic channel, which provide strong gradients. These pole piece structures are magnetized by a magnet outside the microsystem. Since the magnetic reluctance of the pole pieces is smaller than that of the surrounding glass, the magnetic field concentrates. By placing these structures (Figure 3a) around the channel in a zig-zag manner, the gradient constantly changes along the channel.

In the linear regime of the magnetization curve (unsaturated particle), Equation (3) can be written as:

(4) $${\vec{F}}_{mag}=\frac{1}{2\mu_{0}}\chi V_{m}\nabla {\vec{B}}^{2}$$

with $\mu_{0}$ as the permeability in a vacuum, χ as the magnetic susceptibility of the particle relative to the surrounding medium, and $V_{m}$ as the particle’s magnetically active volume fraction. The particle accelerates when the magnetic force (Equation (3)) is stronger than the hydrodynamic drag force (Equation (1)).

Superparamagnetic beads (2.8-µm Dynabeads 10006D, Life Technologies Corporation, Oslo, Norway) are used for the manipulation experiments. These particles consist of a polymer matrix containing nanoparticles which, due to the small size of some nanometers, show superparamagnetic behavior. Magnetic dipoles can be induced by a magnetic field within these particles [25], and chain-like formations are formed while the particles flow through a channel. These particle aggregates disintegrate spontaneously when the field is removed.

Figure 3a shows a simulation (using the open source program FEMM 4.2 in Supplementary Materials) of the magnetic field density between interlocked trident pole pieces along a fluidic channel exposed to the field of a permanent magnet. The strength of the magnetic field periodically changes on both sides of the microchannel (Figure 3b), and the areas with a highly concentrated magnetic flux form a zig-zag-like corridor.

Superparamagnetic beads can be functionalized with various biomolecular substances [26,27]. An antibody functionalization in a similar manner as for the mass enhanced QCM measurement is possible. Since these particles have a large surface-to-volume ratio, a large surface is available for binding or reaction with target analytes while they are flowing through the channel.

### 2.3. Microfabrication of Novel Manipulation System

**Figure 4:**
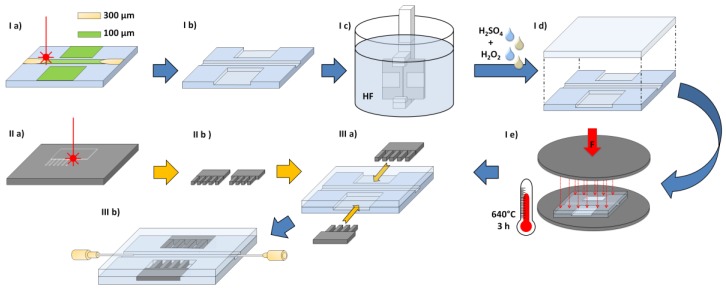
Process for fabrication of a magnetic manipulation system made from glass and a stainless steel foil by fs-laser machining (the final device fabricated with this process is shown in Section 3.2).

**Figure 5:**
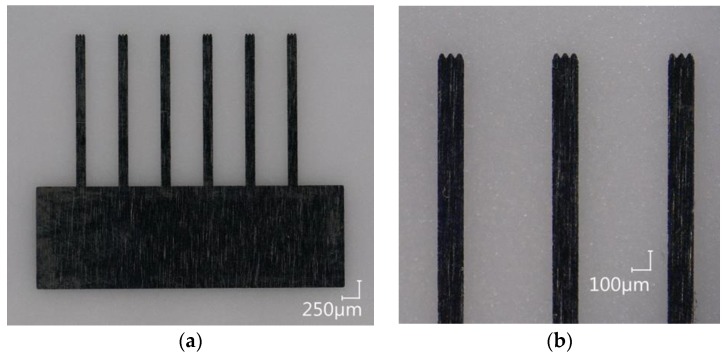
(**a**) Pole pieces inlay for the particle manipulation system. (**b**) Detailed view of three trident pole piece shafts.

The magnetic manipulation system is almost completely fabricated with a femtosecond (fs-) laser system that does not require a clean room environment. A frequency-doubled Yb:KGW-solid-state laser (Light Conversion Pharos, Vilnius, Lithuania) with 515-nm wavelength is used as the laser source. Furthermore, the laser machining system includes a galvanometer scanner system (Scanlab RTC 5, Scanlab GmbH, Puchheim, Germany) embedded into a micromachining platform (Microstruct-C, 3DMicromac, Chemnitz, Germany). In the past, this laser system was already used to fabricate devices for nanoparticle precipitation [28], the analysis of pancreatic islets [29], point-of-care tests from nitrocellulose [30], and shape memory alloy (SMA) actuators made from NiTi material [31]. The fabrication process utilized in this work is shown in Figure 4:

The fluidic channel (I a) and the cavities for the pole pieces are laser ablated 100-µm deep into a glass substrate (Borofloat 33, Schott AG, Mainz, Germany). For the later connection of a cannula, the channel is deepened and widened to 300 µm at both ends. The pole pieces are laser cut from a 50-µm thick stainless steel foil (1.4310, Art-Nr. 80005, H+S Präzisionsfolien GmbH, Pirk, Germany) with the fs-laser system (II a and Figure 5). In principle, it is possible to also use other kinds of materials, since the laser parameters can be adjusted. The fs-laser parameters used in this work are shown in Table 1:

Glass particles that may be generated by the cold ablation process around the channels and cavities are removed by a 60-s dip into hydrofluoric acid (HF) (I c) within a glove box with a fume cupboard. (Safety note: In addition to its ability to dissolve numerous materials, hydrofluoric acid is extremely toxic. It is a contact poison that can interfere with the body’s calcium metabolism, and even the fumes above the etching solution can cause heavy damage to the body or even death).

Afterwards, remaining impurities are removed within an acid-cleaning system by a high-pressure water jet and a mixture of hydrogen peroxide and sulfuric acid. Directly after this cleaning procedure, the glass substrate is pressed onto another cleaned, but unstructured glass lid to achieve a prebond (I d). Since no structures are placed within the glass lid, no wafer alignment is necessary. The prebond package is then placed within a mechanical press, and a load of 10.33 kN is applied (I e). After this step, the whole press is placed within an oven for thermal bonding for 3 h at 640 °C. After bonding, the glass systems are separated by dicing, opening the cavities for the pole pieces. The pole pieces are then inserted from the side (III a) into the tightly fitting cavities. Finally, the pole pieces are glued into the system by an UV-activated adhesive (Photobond GB 368, DELO Industrie Klebstoffe GmbH & Co. KGaA, Windach, Germany). In the same way, the cannulas (PN 7018462, Nordson EFD Deutschland GmbH, Oberhaching, Germany) are connected to the fluidic channel (III b).

## 3. Results

### 3.1. QCM Biosensing Utilizing Mass Amplification

**Figure 6:**
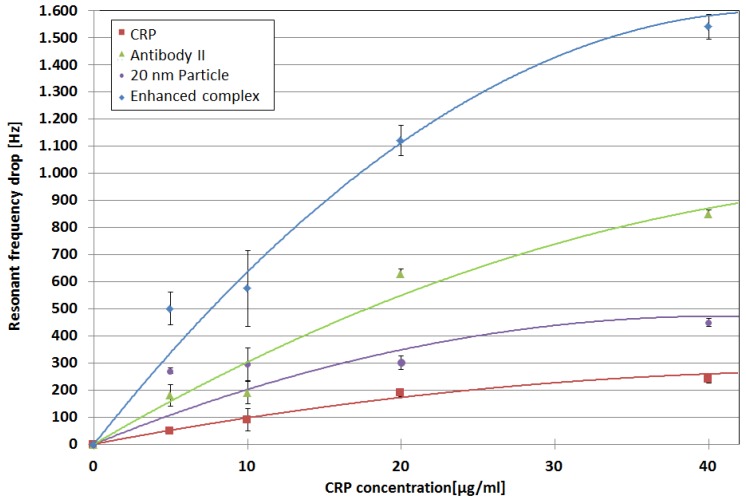
Resonance frequency change for several steps of additional mass up to the complete CRP nanoparticle sandwich. The amount of applied second antibody and nanoparticles is constant for all of the CRP concentrations. Trendlines serve as guide for the eye only.

Measurements analogue to the one described along Figure 2 with a QCM device similar to that shown in Figure 1 were repeated with varying CRP concentrations. The results are summarized in Figure 6:

The step-wise mass enhancement by applying the biotinylated second antibody and the streptavidin-coated nanoparticles leads to according frequency drops for all of the CRP measurements. The measured frequency drops were analyzed by comparing the mean resonant frequency values of the baseline that were developed after the application of a sample with the pre-application baseline. Fluctuations around the old and new baseline mean values were added as error bars in Figure 6. The error bars around the data points for the enhanced complex represent the sum of fluctuations for all of the single components.

For CRP, a linear relation between the applied CRP concentration and frequency drop was found for the range between 5–20 µg/CRP/mL. For higher CRP concentrations, saturation effects start to occur, and the relation between applied CRP amount and resonant frequency drop was no longer linear. While the sensor is able to detect smaller amounts of CRP, 5 µg CRP/mL was chosen as lower measurement limit. Here, the sensor response is clearly visible (already five times higher than noise, while two times higher than noise would already be sufficient) even without additional sandwich components, and hence allows for a good comparison. The actual limit of detection is expected to be lower than 5 µg CRP/mL.

The linear behavior continues for the application of the second antibody, and the signal increases further for the application of the streptavidin-coated nanoparticles. Nevertheless, the impact of the particles on the resonant frequency drop seems to decrease for higher CRP concentrations. Since approximately 8.6 × 1012 particles are applied in one 100-µL injection, there are enough particles to saturate the available binding sites. Another explanation might be steric hindrance, and hence a reduced ability to attach to the remaining free binding sites for higher CRP concentrations. In the negative control experiment, no interactions of the particles with the QCM in absence of the second antibody and CRP were found, and hence, a non-specific binding of particles can be excluded. In total, the application of the antibody-particle complex leads to a signal amplification by a factor of five compared to measurements with a sole CRP attachment.

Another advantage of the continuous operation provided by the microfluidic environment over incubation approaches is the possibility to calibrate and apply numerous samples directly after another in a rapid succession. If the mass amplification is already sufficient without the attachment of additional particles, the volume flow can be increased from 0.05 µL/s to 0.5 µL/s, increasing the measurement speed by a factor of 10. The low volume flow of 0.05 µL/s is only necessary to reduce the drag force of the flow acting on the particles.

### 3.2. Magnetic Bead Manpulation

**Figure 7:**
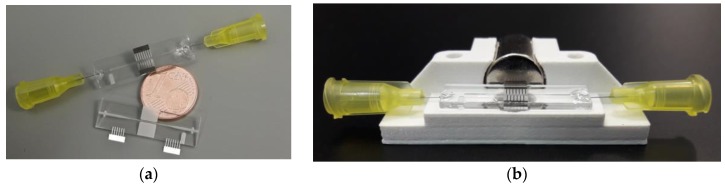
The microfluidic bead convection system. (**a**) The glass channel and pole piece inlays in comparison to a one-cent coin, as well as the assembled device. (**b**) The system with a cylindrical permanent magnet placed in a three-dimensional (3D) printed holder.

**Figure 8:**
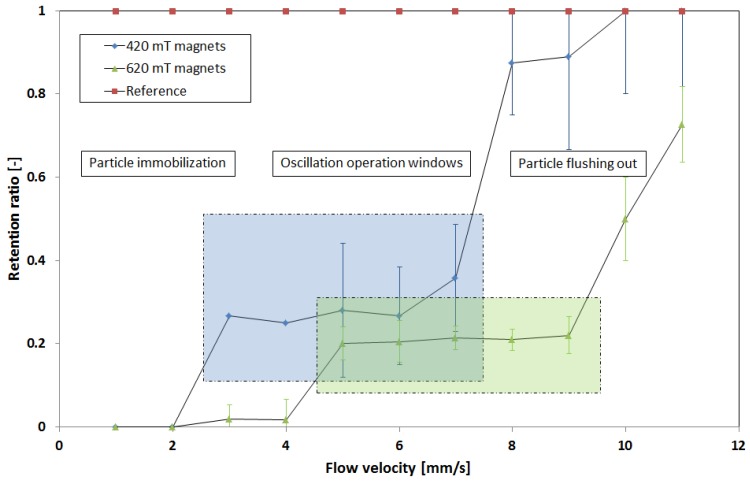
The retention ration as a function of fluid flow velocities. Typical operational characteristics for the particle convection device equipped with two magnets for varying flow velocities are indicated. The squares represent the oscillating operation mode for the two different magnet pairs. Before reaching the corresponding flow velocities, particles get immobilized near the pole pieces, while for higher flow velocities, the particles leave the system without showing any signs of induced fluctuation.

For operation, at least one (two are also possible) permanent magnets have to be located close to the pole pieces on one side of the system; then, the system is ready for operation. The final microsystem is shown in Figure 7:

Typical operation windows were identified earlier [10]. Here, we introduce the retention ratio parameter to characterize a system with pole pieces and two attached magnets, as shown in Figure 7. The velocity of the particles without attached magnets is measured to provide a reference. The retention rate is defined as the ratio of the particle velocity with magnets over the particle velocity without magnets. A retention ratio of 1 means that the particle transport is not affected by the magnetic field, whereas a retention rate of 0 implies the immobilization of the particles. The retention rate allows a comparison of the particles’ behavior for different magnets and flow velocities. For the determination of mean values, the experiments were repeated several times. The error bars provided in Figure 8 indicate variations due to particle cluster formations. The particles start to form chains and agglomerates, and the force balance between the hydrodynamic drag force and the magnetic force changes in the process. Longer particle chains or clusters experience lower drag forces compared with their magnetic force, and hence move faster through the liquid to the pole pieces. As a consequence, not all of the particles are equally fast.

**Figure 9:**
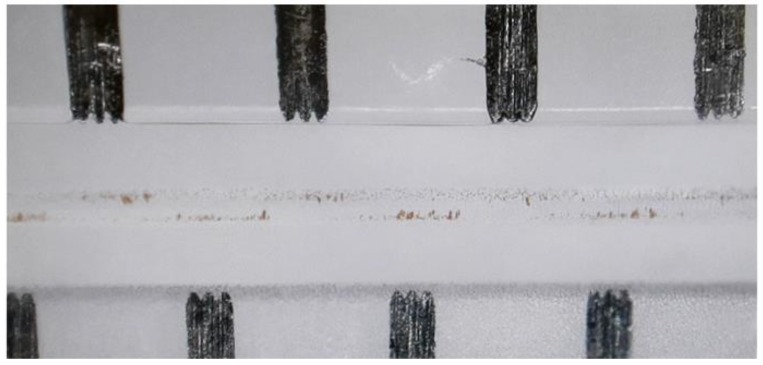
Close-up of the fs-laser fabricated pole pieces along the microfluidic channel with immobilized superparamagnetic beads.

**Figure 10:**
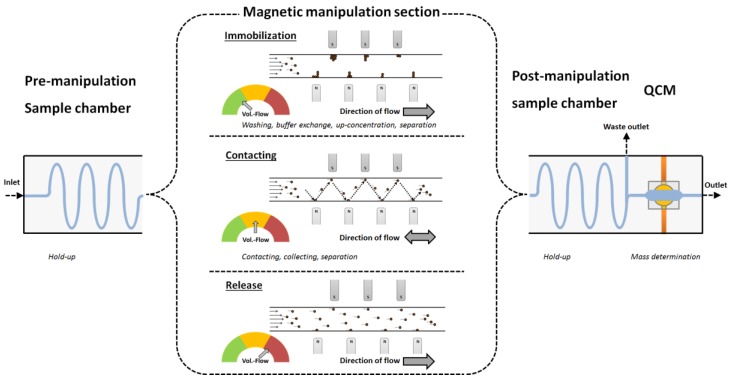
Operation mode concept with achievable unit operations of the magnetic manipulation system in combination with an attached QCM sensor.

The balance between magnetic force and hydrodynamic drag force was controlled by the volume flow, and hence, the velocity of the fluid and particles (2.8 µm Dynabeads 10006D, Life Technologies Corporation). It was observed that for low-volume flows, the magnetic force dominates, and particles are immobilized near the pole pieces on the channel walls. When the flow rate was increased and the drag force started to overcome the magnetic force, particles began to follow the flow. They were then deflected by the magnetic field, following the magnetic gradient in a zig-zag-like manner between the pole pieces on both sides of the channel. For higher flow rates, the particles moved faster, and were only slightly deflected by the magnetic field. For even higher flow rates, no attraction was visible anymore, because the residence time of the particle within the microchannel was too short. Two magnet pairs with different strengths (420 mT and 620 mT) were used to manipulate the particles within the channel. As a result, the operation window for oscillation (indicated by the blue and green squares in Figure 8) shifted slightly. The stronger magnet was able to immobilize particles for slightly higher flow rates, and in turn, its oscillation operation window also shifted a little to higher flow rates. Nevertheless, it showed the same overall operation behavior. In Figure 9, a close-up of the particles immobilized at the channel walls near the pole pieces is shown:

According to its operation characteristics, three regimes can be identified for implementation of this subsystem within a lab-on-a-chip. These include the immobilization or separation of particles, the fishing-like contacting of the particles and the sample in a controlled zig-zag manner, and the particle release. The operational regimes can be selected by the pump rate (see Figure 8), and do not require the active control of the magnets. The automation of this system is possible with little effort. This subsystem can also be utilized for washing, up-concentration, or a buffer exchange when the critical components of a sample bind to the particles that are applied. For this, the sample has to be pumped back and forth through the channel. The active motion of the particles decreases the diffusion paths and accelerates the binding of molecules to the particles. This process is independent of the sample size, and can be carried out endlessly. When the relevant component is bound to the sample, the particles can be immobilized within the system again or released for further analysis. This concept is further summarized in Figure 10:

## 4. Discussion and Outlook

In this work, two possible methods for improving the measurement of biological samples utilizing particles were presented. These methods were: direct mass enhancement with particles on a QCM, and the convective pre-preparation of the sample by particle based up-concentration, including the possibility of separating the analyte from other sample components or reducing the sample size. The utilization of superparamagnetic particles allows the enhancement of the signal response of a QCM by up to a factor of five. As a result, it enables it to measure concentrations of the target analyte that were not measureable before. The simple manipulation system allows the further utilization of the necessary superparamagnetic particles for different sample preparation steps controlled by the flow rate. For the magnetic field source, only one static, external permanent magnet is necessary, as it allows for manipulation in a seesaw-like manner without the need for an additional field source to generate a magnetic gradient. This makes the overall setup very cheap compared with other magnetic manipulation setups. Since the full potential of both methods lies within their combination, a concept for a possible lab-on-a-chip system is presented in the following as a possible outlook. The aim of the combination into one device is to implement all of the sample preparation steps and target analyte quantification into one microfluidic chip while providing measurement results in a shorter time frame than commercial enzyme-linked immunosorbent assay (ELISA) tests. By adapting the flexible fs-laser process to the new design, fabrication of the whole device is expected to be possible in glass, which would allow for a potential cleaning and regeneration of the sensor system, and hence its reuse, instead of a disposable-centered approach.

To achieve an optimal combination of the two systems into one lab-on-a-chip, the operation conditions have to be synchronized concerning their operational parameters and the particles used within the overall system. The main control parameter is the flow rate, which can be directly regulated by the pump. Since the performance of separation, up-concentration, and manipulation steps is necessary before the particle-enhanced measurement, the particle convection unit will be located before the QCM sensor. The flow rate for performing the different operations within both units will be adjusted by the pump separately after another.

Nevertheless, the particle selection requires a compromise. This work utilizes streptavidin-coated superparamagnetic particles with a cross-section of 20 nm for attachment to the QCM, while bigger particles are favorable for the particle manipulation system, because the achievable manipulation forces are higher. An attachment of 1.08-µm superparamagnetic beads (Dynabeads MyOne Streptavidin T1, Life Technologies Corporation) to the QCM was tested, but it did not show as high mass amplification as the 20-nm particles. In contrast, the 2.8-µm big beads that were used for the magnetic manipulation experiments within the microchannel were not suited for this at all. These beads tended to sediment within the injection valve and the tubes before reaching the QCM.

For beads/particles that were directly injected into the manipulation system, this behavior was not as critical, since the particles could still be stimulated to roll over the channel floor in a zig-zag-like manner. According to the manufacturer of the 2.8-µm and 1.08-µm beads, the 1.08-µm particles were optimized for slow sedimentation. When the 1.08-µm beads were manipulated, they also followed the path generated by the pole pieces, and did not sink while in the system. Nevertheless, magnification of the optical microscope that was used reached its limit, and did not allow for precise evaluation/analysis.

Despite this, an optimal particle size for both systems would be within the sub-µm range. As discussed above, this size strongly depends of the physical properties of the samples analyzed as well as on the operational parameters of the systems, which in turn allow for the use of a variety of different particle sizes. For the QCM device, the smallest particles possible are favorable. According to the review of Skládal [8], all kinds of particles with sizes between 30 and 970 nm are already successfully used for mass amplification on a QCM sensor. This indicates some degree of freedom when it comes to particle selection for QCM mass amplification, and allows the selection of particles with a size that is also suited for manipulation.

Since the QCM is embedded into PDMS, and the particle manipulation system is made from glass, these two materials are preferred. The advantages of PDMS are that it is cheap, and can be fabricated by soft lithography very rapidly when a negative mold is available. It is biocompatible, and since it is its flexible, the quartz sensor crystal can be inserted into its associated cavity without any fitting problems based on fabrication imperfections concerning its contour accuracy [32]. However, storage of SAM-prepared QCMs for over two weeks is not advisable, due to reactivity losses and because an operation over a longer period of time increases the risk of cross-contamination between different patients’ samples. As a result, the original QCM sensor was designed to be disposable. Other risks associated with the utilization of PDMS involve possible swelling and the risk of proteins getting embedded into the PDMS, which in turn could lead to sample loss, false quantification results, or even cross-contamination. This can be prevented by different approaches, such as hydrophilization of the PDMS for example, as was already investigated in Demming et al. [33].

Another problem concerns the leakage tight bonding between the pole pieces and the corresponding microfluidic channel. While it is possible to produce a leak-tight system, most of the systems that were fabricated in the past suffered from leakage from the fluidic channel into the cavities containing the pole pieces.

The fabrication of a system totally made from glass could be largely carried out with the fs-laser system. Only the quartz crystal disc would still involve a photolithography approach; hence, a clean room is still necessary for fabrication. The bonding between glass and quartz should be possible since the thermal expansion coefficients of glass and quartz can be adjusted by the selection of a convenient type of glass. The melting points of chrome and gold, the electrode materials, lie at approximately 1.907 °C and 1.064 °C, respectively, and hence are not reached while bonding. The pole piece inlays, as well as electrical contacting by the conductive varnish (Eccobond 59 C, Emerson & Cumning, Geel, Belgium) of the quartz crystal, are done from the side after the assembly of the quartz crystal and the top and bottom cover. This step is independent of the material choice. The biggest advantage of glass over PDMS is its chemical durability. This allows the system to be cleaned and for the surface activation to be removed before it is renewed and the system can be used again. The risk of particle attachment can be reduced by further smoothening the lasered channel. The impact of changing HF-dip times on fs-lasered microchannel smoothening was already investigated in Erfle et al. [28].

The most straightforward approach to combine both systems is to place them directly in a row on one chip. Nevertheless, if the samples have to be pumped repeatedly in between the pole pieces, there might be the need for some hold-up channels before and after the manipulation channel. If the volume of the sample is too big, also at least one valve is necessary. The hold-up channels are already indicated in Figure 10, allowing for a change in flow parameters for washing, up-concentration, or a buffer exchange before the sample reaches the QCM. Since the hold-up channels can be fabricated with the fs-laser, similar to the rest of the fluidic channels on the chip, their integration is possible without any further effort.

The implementation of a valve is more complicated. While there exist numerous different approaches to realize microvalves [34,35], nearly all of the designs involve moving parts, such as lids or membranes and/or other additional components such as wires for the realization of piezoelectric or electroosmotic principles, hydrogels, or even additional channels for pneumatic actuation principles. The need for at least one additional valve between the magnetic manipulation channel and the QCM could be bypassed by operating the device with two, instead of one, pumps in sucking mode from the outside. One would be positioned at the waste outlet between the manipulation channel and the QCM, and one would be positioned behind the QCM. Depending on which pump is operating, the sample solution would be transported out of the waste outlet or over the QCM.

**Figure 11:**
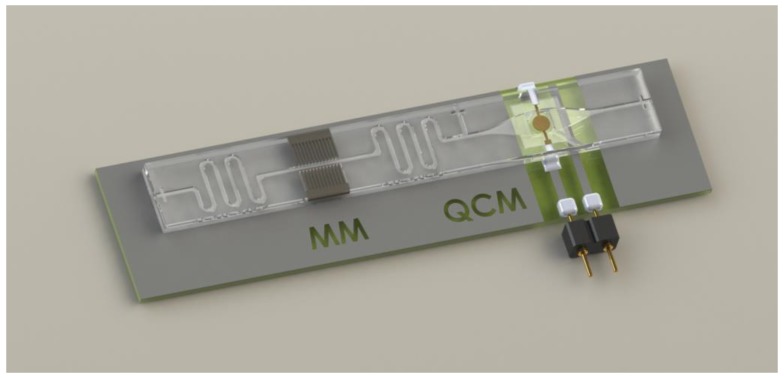
Concept study for the combination of a magnetic manipulation system with fs-laser machined pole pieces and a microfluidic QCM sensor into one lab-on-a-chip system fabricated in glass.

A possible concept for the combination concept for both systems is shown in Figure 11:

Based on the proven functionality of the individual units, our next step will be the fabrication of a combined reusable system in glass to achieve a lab-on-a-chip that combines the sensitivity enhancement by integrated convective up concentration and mass amplification with superparamagnetic beads.

## Figures and Tables

**Table 1:** Laser process parameters for structuring glass and stainless steel foil substrates.

Parameter	Glass	Stainless Steel Foil
Wavelength	1030 nm	515 nm
Repetition rate	100 kHz	100 kHz
Laser power	5.26 W	1.67 W
Pulse duration	238.3 fs	238.3 fs
Scan speed	750 mm/s	750 mm/s

## Supplementary Materials

The measurement data used for the generation of Figure 2, Figure 6 and Figure 8 and the FEMM 4.2 simulation files for generation of Figure 3 are available online at http://www.mdpi.com/2072-666X/9/4/194/s1 as supplementary material.
